# The Role of ESS2/DGCR14: Is It an Essential Factor in Splicing and Transcription?

**DOI:** 10.3390/ijms26094056

**Published:** 2025-04-25

**Authors:** Ichiro Takada, Shinya Hidano, Tohru Nakagawa, Shinichi Nakagawa, Makoto Makishima, Sayuri Takahashi

**Affiliations:** 1 Department of Urology, The Institute of Medical Science, The University of Tokyo, Tokyo 108-8639, Japan; 2 Division of Biochemistry, Department of Biomedical Sciences, School of Medicine, Nihon University, Tokyo 173-8610, Japan; makishima.makoto@nihon-u.ac.jp; 3 Department of Urology, Teikyo University School of Medicine, Tokyo 173-8605, Japan; nakagawat@med.teikyo-u.ac.jp; 4 Department of Immune Regulation, The Research Center for Hepatitis and Immunology, Research Institute, National Center for Global Health and Medicine, Chiba 272-8516, Japan; lb-21hidano@hospk.ncgm.go.jp; 5 RNA Biology Laboratory, Faculty of Pharmaceutical Sciences, Hokkaido University, Sapporo 060-0812, Japan; nakagawas@pharm.hokudai.ac.jp; 6 Department of Urology, The Faculty of Medicine, The University of Tokyo, Tokyo 113-8655, Japan

**Keywords:** transcription factor, transcriptional coregulator, spliceosome complex, 22q11.2DS, cancer, cell differentiation and proliferation, immune system, neuron

## Abstract

ESS2 (ess-2 splicing factor homolog, also known as DGCR14 or DGS-I) is a member of the deletion gene cluster in the 22q11.2 deletion syndrome (22q11.2DS, also known as DiGeorge syndrome or CATCH 22 syndrome). The ESS2 gene is not part of a gene family, and the coded protein has a coiled-coil structure (Es domain), which is conserved from yeast to humans. Recent studies have shown that ESS2 is involved in splicing C and C* complex, but other interactants, such as transcription factors and U1 snRNP, are also reported. Although the molecular mechanism is still under investigation, ESS2 plays a pivotal role in cell differentiation and proliferation. ESS2 knockout mice show embryonic lethal in the early stage, and recent studies show the association of ESS2 with cancer, autoimmune disease, and neurodevelopmental disorders. ESS2 can regulate mRNA splicing and transcriptional activity through interactions with other proteins, and ESS2-dependent gene expression regulation seems to be cell type-selective. In this review, we summarized the cloning history and functions of ESS2, including recent findings.

## 1. Cloning of ESS2 and Its Structure

### 1.1. Cloning of ESS2 (DGCR14)

In humans, the *ESS2* gene was first cloned as an expression sequence tag located in the 22q11.2 locus, which is related to the 22q11.2 deletion syndrome (22q11.2DS; also known as DiGeorge syndrome or CATCH 22 syndrome) by two groups independently [1,2]. 22q11.2DS is a disorder caused by a small deletion located near the middle of chromosome 22, specifically designated q11.2 [3,4,5]. Common signs and symptoms of this syndrome include heart abnormalities, cleft palate, distinctive facial features, and schizophrenia. In addition, 22q11.2DS patients are often immunodeficient and experience recurrent infections, and some patients develop autoimmune disorders, including rheumatoid arthritis and Graves’ disease [6].

After defining the DiGeorge syndrome critical region using FISH [7] and isolating the region as cosmid and YAC clones, researchers performed cDNA cloning from this region and identified ESS2 as one of the cDNA clones. Baldini’s group, for instance, identified six cDNA clones from the 22q11.2 cosmid and named these cDNAs Expressed Sequences (*ES*)*1*, *ES2*, *ES3*, *ES4*, *ES5*, and *ES6* [2]. Among them, *ES2* was the cDNA corresponding to *DGCR14* and was finally named *ESS2* (*Ess-2 Splicing Factor Homolog*). According to the literature and information in GenBank, *ES1* was *IDD*, *ES3* was *CLTCL*, *ES4*, and *ES5* were fragments in the *DGCR2* gene, and *ES6* was *TUPLE1*. Budarf’s group identified at least 11 transcription units encoded in the minimum DiGeorge syndrome critical region (MDGCR), and *DGS-I* (*DiGeorge Syndrome Gene I*) corresponded to *ESS2* [1].

After the identification of *ESS2* in humans, *ESS2* homologs were cloned in *Caenorhabditis elegans* (F42H10.7) [8] and *Mus musculus* [9]. From the beginning of cloning, tissue-specific expression of *ESS2* in humans was also confirmed by Northern blotting [2]. The major transcript of *ESS2* was estimated to be approximately 7 kb, and an approximately 2 kb band was present in every tissue tested, which suggested alternative splicing. Northern blotting of *ESS2* in human fetal tissues (brain, lung, liver, and kidney) showed a 2 kb band in all four tissues tested, but no 7 kb band was detected. Other groups also examined Northern blots and showed that a 5.2 kb fragment was only observed in heart and muscle tissues, and a 1.7 kb transcript almost uniformly appeared [1,9]. In mice, two *ESS2* transcripts of ~2 and ~3 kb were identified in early embryogenesis (E7, E11, E15, and E17) [10]. In adult mice, *ESS2* was expressed in all tissues tested (heart, brain, spleen, lung, liver, skeletal muscle, kidney, and testis) and was highest in the testis and the brain. The two *ESS2* transcripts’ expression levels varied among the tissues and also within the same tissue [10]. These data may indicate the existence of tissue-dependent *ESS2* splicing variants, but there are no future studies about these variants. No ncRNAs derived from the *ESS2* locus have been reported.

**Figure 1:**
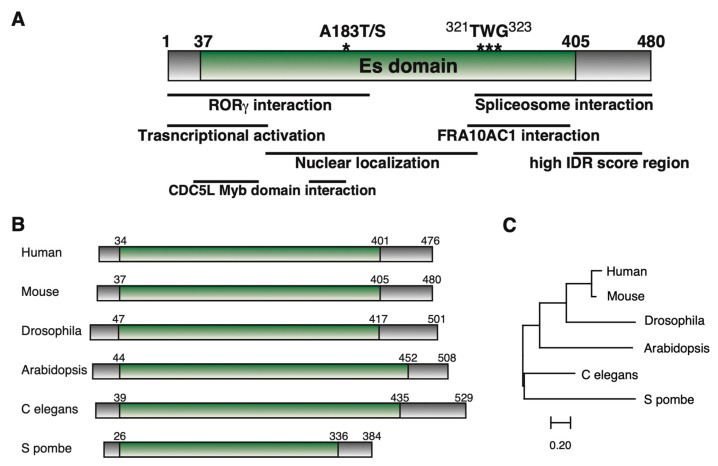
(**A**) ESS2 protein domain structures. We summarized the domains of ESS2 from references corresponding to murine ESS2. * means the location of A^183^ and *** means the location of ^321^TWG^323^. (**B**) Compare ESS2 between species described in this review. (**C**) Phylogenetic tree from DNA sequences coding Es domain by using MEGA11 (https://www.megasoftware.net/ accessed on 5 April 2025).

Although *ESS2* cDNAs have been cloned in various species since then, none of them belong to a gene family, and only one gene exists in each species. Most of the human 22q11.2 deletion region was conserved in the mouse 16qA3 region, but in zebrafish, fruit flies, and nematodes, each gene was located on a different chromosome [11]. The number of amino acids coded for by the *ESS2* cDNAs ranged from 384 (*Schizosaccharomyces pombe*) to 631 (*Tyrophagus putrescentiae*), although most species coded for about 470-500 amino acids. All ESS2 gene products had unique Es domains consisting of coiled-coil domains for interacting with other proteins, as mentioned in this review (Figure 1A–C).

Furthermore, there were several genes containing Es domains identified from databases. In *Mycoemilia scoparia*, an ATP-dependent 3′-5′ DNA helicase contained an Es2 domain (KAJ1920831.1). This Es domain had low homology (~30%) with other ESS2 homologs, including a hypothetical zinc-ring finger-containing protein (KAF9320509.1) in *Podila horticola*. In *Labeo rohita*, 883 amino acids of STAM-binding protein-like A were registered (KAI2659573.1), which included the USP8 dimerization domain and MPN_AMSH_like domain, as well as the Es2 domain. However, this STAM-binding protein-like A protein was missing a part of the Es domain (about 32 amino acids), and ESS2 was also present (XP_050977276.1) with 93% homology in *Labeo rohita*. Genes with this type of Es domain have not yet been found in other species.

### 1.2. ESS2 Interactants

ESS2 has also been identified by several proteome projects. In splicing complex purification and proteome analysis, ESS2 has been identified as a peripheral factor of the C complex in *Drosophila* [12] and humans [13,14]. Recent studies showed that spliceosome complexes included numerous proteins referred to as peripheral factors, in addition to the core complex components [15].

ESS2 is also involved in spliceosome activation during step 2 of splicing (C* complex) [16]. Pre-mRNA splicing involves two sequential reactions: branching and exon ligation. The C complex undergoes branching, and the C* complex executes exon ligation by catalyzing the 3′-OH group of the 5′ exon, which then attaches to the 3′ splice site [17]. In addition to ESS2, FAM32A [18], SDE2, CACTIN, PRKRIP1, NKAP, TLS1, CXORF56, FAM50A, PPIL3, PPIG, NOSIP, GPATCH1 and the RNA helicases DDX41 and DHX35 were also involved in the spliceosomal C* complex. From single-particle cryo-EM of the human PM5 C* complex, researchers determined that ESS2 was located near NOSIP. Furthermore, two α-helical regions (amino acids 32 to 97 and 140 to 177) of ESS2 contacted the CDC5L Myb domain. These two α-helical regions also interacted with the N-terminus of the long α-helix of SKIP, which binded PRP22. Although ESS2-dependent regulation of splicing fidelity and exon selectivity have been reported in some conditions [19,20,21,22], the molecular mechanism of ESS2 during the splicing process is still under investigation.

In addition, interactions between transcription factors and ESS2 have been reported. In MYC, an interactome by IP-MS using MYC homology boxes (MBs) showed multiple interactants, including ESS2 [23]. The SP7 transcriptional factor, which belongs to the Sp family of zinc-finger transcription factors, also identified ESS2 from IP-MS data [24]. We also identified ESS2 as a nuclear receptor for retinoic acid-related orphan receptor-gamma/gammat (RORγ/γt) interactants in a T cell-like cell line (68-41 cells) using affinity purification of RORγ and mass spectrometry [25]. Interestingly, ESS2 also interacted with NR4A1 from IP-MS data [26], which belongs to the nuclear receptor superfamily [27,28]. Finally, another nuclear receptor, TR2 (NR2C1), also shows the interaction with ESS2 by IP-MS data [29].

**Figure 2:**
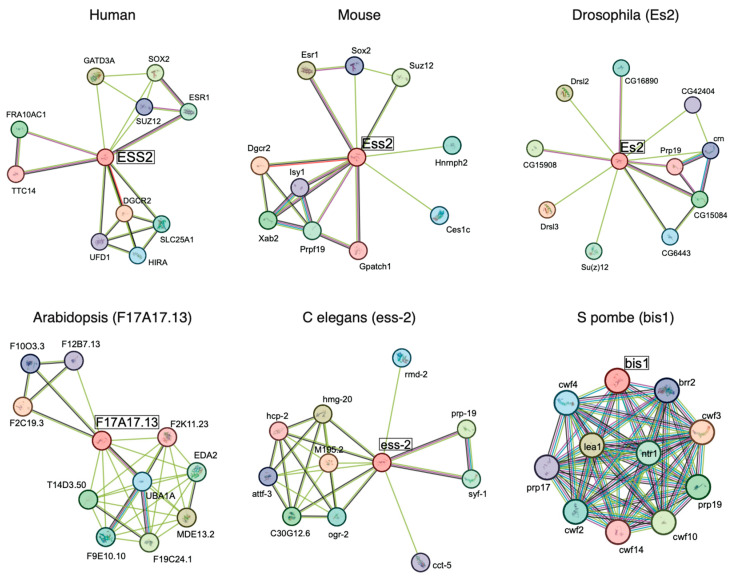
ESS2 interactome from STRING interaction network.

These results show that ESS2 can be associated with RNA processing complexes and transcriptional factors. Other interactome results are shown, such as those in the NIH database (https://www.ncbi.nlm.nih.gov/gene/8220 accessed on 3 July 2023). In the STRING interaction network (https://string-db.org/ accessed on 12 April 2025), ESS2 interactants are different from species (Figure 2). It is interesting to note that most of the interacting factors in yeast are splicing-related factors, whereas interactions with transcription factors (Sox2, ESr1) and polycomb factors involved in gene silencing (Suz12) are also observed in mammals. These data suggest that the *ESS2* gene may have acquired diverged functions during the evolution.

### 1.3. Mutations of ESS2 and Disease

The association between *ESS2* gene mutants and disease has been reported. One SNP in *ESS2* (rs62223875), which is the missense mutation A179T (A183 in mice), was identified as a suicide-associated gene [30], although the functional changes caused by this mutation were unclear. In some cases, promoter mutations of the *ESS2* gene caused different transcriptional activities. Haplotype promoter activity was measured using a transfection luciferase reporter assay in HEK293T (human embryonic kidney) and TE671 (human rhabdomyosarcoma) cells [31]. In HEK293T cells, either the g.2284475C4T polymorphism or g.2284644G4A (or both combined) affected transcription two-fold. In TE671 cells, either g.2284535C4T or g.2284530A4T (or both combined) affected transcription two-fold. These results showed the cell dependent-promoter activity of ESS2 and the possibility for different expression systems among cell types.

Mutations in the *ESS2* promoter region have also been reported to be associated with schizophrenia [32]. These results showed significant evidence for preferential transmission of the promoter variants of *ESS2* (rs1936950 (A>T) and rs1936951 (G>A)) in schizophrenic males. The presence of mutations at multiple sites suggested that changes in mRNA expression were due to altered transcription factor binding, but the details were unclear.

A study of genetic defects in Korean patients with aortic dissection (AD) showed a significant decrease in *ESS2* mRNA [33]. Since 22q11.2 deficiency also causes cardiac abnormalities, it was unclear whether this result was due to a defect in the entire 22q11.2 region or in *ESS2* alone. Specifically, this could be the result of a deletion or mutation of the entire 22q11.2 region or a single *ESS2* mutation.

Overall, these studies demonstrate the possibility for ESS2 to become a new therapeutic target for related diseases.

## 2. Function of ESS2

Although ESS2 was discovered about 30 years ago, its molecular mechanism is still under investigation. Its nuclear localization [10] and the early lethality of systemic *ESS2* mutant mice [34] show its importance in nuclear events such as transcription and splicing, but the precise cause of lethality is still unknown. However, *ESS2* has been isolated and its mutants have been generated in several model organisms, and some aspects of its function have been elucidated. ESS2 target genes and the molecular mechanism of *ESS2*-dependent lethality are still under investigation.

### 2.1. ESS2 Function in Yeast

The yeast *ESS2* homolog has been identified as an interactant of ISH1 (induced stationary phase protein), which is the nuclear envelope protein, and is named *BIS1* (binds to Ish1). Overexpression of BIS1 causes a cell elongation phenotype, and *BIS1*-deficient strains have reduced viability [35]. BIS1 has also been shown to change localization during the cell cycle, localizing to the nucleus and the mitotic spindle microtubules and associating with the nuclear envelope. As shown in two-hybrid experiments, BIS1 interacted with some proteins, such as arginine methyltransferases (PRMT5), splicing subunits, and nuclear envelope factors. PRMT5 has been reported to be involved in T cell development [36] and cancer proliferation [37] in mammals. Although the homology between BIS1 and ESS2 is not high, testing the interactions between ESS2 and mammalian homologous genes will be an interesting avenue for future research.

### 2.2. ESS2-Dependent Splicing Regulation in Arabidopsis

*ESS2* has been isolated in one of the Pre-mRNA Splicing Mutants Screenings in *Arabidopsis thaliana* [21]. Researchers developed a novel splicing-dependent GFP reporter system in Arabidopsis to carry out this forward genetic screen. In this system, an intron-containing GFP reporter gene present in a wild-type ‘Target’ (WT T) line exhibited variable levels of GFP expression depending on the splicing pattern of its pre-mRNA. By screening for changes in GFP fluorescence in seedlings derived from chemically mutagenized seed, they retrieved nine hgf (Hyper GFP) mutants and seven gfw (GFP weak) mutants. Among these mutants, *dgcr14-1* (Q80*) and *dgcr14-2* (W365*) have been identified as hgf mutants. They showed that these mutations promoted splicing efficiency but had no effect on *Arabidopsis* growth. Since Q80* contains few or no Es domains, it appeared that *Arabidopsis* development was possible with only the N-terminus of ESS2. However, it was not clear whether *Arabidopsis* could develop without ESS2. This work suggested that the presence of a splicing complex interaction region at the C-terminus contained W365. Thus, *dgcr14-2* (W365*) mutants may prevent interactions with the splicing complex.

Recent studies showed the importance of ESS2 in NaCl stress-dependent splicing in *Arabidopsis thaliana* [22]. Researchers generated *Arabidopsis* with a mutant *ESS2* homolog gene (*AtDGCR14L*; *Arabidopsis thaliana* DiGeorge Syndrome critical region 14-like) and generated overexpressed *AtDGCR14L* in this mutant. Interestingly, when raised with 200 mM NaCl, the *AtDGCR14L* mutant showed significantly reduced viability, and overexpressed *AtDGCR14L* rescued this lethal phenotype. Endogenous AtDGCR14L is expressed in the early phases of NaCl stress and regulates the gene expression of numerous genes. They performed RNA-seq and investigated the salt-stress dependent genome-wide pre-mRNA splicing events. In the *AtDGCR14L* mutant, they identified 684 differential alternative splicing (DAS) events in 552 unique genes before salt-stress treatment and 838 DAS events in 699 unique genes after salt-stress treatment. In contrast, *AtDGCR14L* overexpression only had 147 DAS events in 126 unique genes before salt-stress treatment, and 114 DAS events in 96 unique genes after salt-stress treatment were identified. These results clearly show the importance of ESS2 in pre-mRNA splicing events. Particularly, they found AtDGCR14L-dependent splicing defects in the *SWI3A* gene, one of the key factors for SWI/SNF chromatin remodeling in *Arabidopsis thaliana* [38]. Furthermore, they identified a TWG motif based on amino acid sequence homology of ESS2 with other species. This sequence was included within the IDR (intrinsically disordered regions) of the C-terminus in ESS2, but overexpression of this sequence variant did not rescue the *AtDGCR14L* mutant phenotype. In mammalian, several reports show the stress-inducible splicing alteration [39,40]. Because ESS2 localization in the nucleus is similar to TDP-43 [40,41], the relationship between ESS2 and TDP-43 in mammalian splicing will be a subject for future study.

### 2.3. ESS2-Dependent Mutated mRNA Splicing Regulation in C. elegans

In *C. elegans*, *ESS2* was identified as a dlk-1 splice acceptor mutation and suppressor of rpm-1, which belongs to the ubiquitin E3 ligase family and is involved in axon patterning and synaptic development [19]. In *C. elegans*, mutations in rpm-1 caused axon overgrowth and abnormal presynaptic morphology without major behavioral defects. However, when combined with mutations in presynaptic assembly factors syd-1 or syd-2, the result was severe locomotor deficits and impaired synapse formation. Researchers screened *rpm-1* suppressor genes and identified the DLK-1 MAP kinase pathway as a negative regulator of RPM-1. DLK-1 is a MAPKKK activating pathway that includes mkk-4 (MAPKK), pmk-3 (MAPK), and mak-2 (MAPK-activated kinase). This pathway activates the transcription factor CEBP1 and regulates synaptogenesis and axon morphogenesis. RPM-1 ubiquitinates and degrades DLK-1 proteins and represses the DLK-1 pathway [42]. When the *DLK-1* gene is mutated in the splice acceptor site, *ESS2* deficiency leads to significantly reduced mRNA expression levels of the *DLK-1* gene. This result may suggest that ESS2 excludes mutated splicing when in normal conditions. This study provides novel insights into how the PHR family regulates synaptogenesis and mRNA splicing in a context-specific manner. Furthermore, ESS2 interacting factors in *C. elegans* are published in STRING (string-db.org accessed on 12 April 2025), including splicing complex components such as prp-17 and prp-31.

### 2.4. ESS2 Function in Drosophila

In other studies, which used RNAi screens to identify regulators of *Drosophila melanogaster* fat cells, *ESS2* has been reported as a gene that reduced fat cells, and is therefore considered a lean gene [43]. Fat cells are a cell type contained within the fat body and are responsible for the regulation of lipid metabolism in insects. The transcription factors that regulate lipid metabolism also have many mammalian homologs, such as SREBP and FOXO [44]. The phenomenon in *Drosophila* fat cells is interesting because ESS2 has not been shown to play a role in lipid metabolism in mammals. Because ESS2 has also been identified in the purification of the spliceosomal C complex in *Drosophila* [12], functional similarities in ESS2 between vertebrates and invertebrates were expected, although the analysis is still in progress. Further analysis, including on the transcriptional regulation by ESS2, is warranted.

### 2.5. ESS2 Function in Mammals

#### 2.5.1. ESS2 Function in Neurons

Abnormalities such as mental retardation are common in DiGeorge’s syndrome. ESS2 has also been reported to function in the nervous system, including SNPs that have been associated with suicide [30] and schizophrenia [32], as mentioned in 1.3.

It has also been reported that the ESS2-dependent induction of protein kinase-R-like Endoplasmic Reticulum Kinase (*PERK*) mRNA expression in dopaminergic neurons differs in 22q11.2DS patients’ iPS cells [45]. Arioka et al. (2021) conducted a semiquantitative proteomic analysis in differentiated neurons in both control and 22q11.DS patients’ derived iPS cells. They identified 272 differentially expressed proteins. Furthermore, KEGG pathway analysis revealed that the most altered pathway in the 22DS group was ‘Protein processing in ER’, which is responsible for the ER stress response and protein quality control. PERK is an ER stress regulator and exhibits lower expression in neurons from 22q11.2DS patients’ iPS cells. They found that *ESS2* RNAi significantly reduced PERK protein levels, although whether ESS2 regulates the transcription or splicing expression of *PERK* mRNA is unclear. Regardless, these results show the importance of ESS2 in ER stress.

In addition, a recent AAV-based CRISPR gRNA screening of 22qDS11.2-related genes in neurons showed that ESS2 acted as a transcriptional regulator in interneurons, superficial-layer neurons, and deep-layer neurons [46]. AAV screening of the genes in the 22q11.2DS deletion region led to the isolation of *ESS2* (*Dgcr14*) as an important factor for transcriptional regulation along with *Dgcr8*, *Gnb1l*, and *Ufd1l*. It has also been demonstrated to regulate gene expression differently from the other three factors, particularly in the regulation of transcription in interneurons. Among genes related to ESS2-dependent regulation of splicing, the researchers found constituents of the serine and arginine protein family (*Srrm2*, *Srsf1*, *Srsf2*, *Srsf5*, *Srsf6*, and *Srsf11*) that are essential for spliceosome assembly, as well as constituents of the heterogeneous nuclear ribonucleoproteins family (*Hnrnph3*, *Hnrnpm*, and *Hnrnpu*) that are involved in pre-mRNA processing, mRNA transport, and metabolism. Furthermore, an analysis of the upregulated genetic program showed disruption of chromatin binding and organization, which included dysregulation of genes from the chromodomain-helicase-DNA-binding family (*Chd1*, *Chd3*, *Chd6*, and *Chd8*), topoisomerases (*Top1* and *Top2b*), and *Setd5*, a gene that regulates chromatin structures to control RNA elongation and splicing.

Interestingly, some studies showed that ESS2 is associated with FRA10AC1, one of the spliceosomal C complex peripheral factors [13,47]. This interaction is confirmed by immunoprecipitation assay. Biallelic *FRA10AC1* variants cause a disorder with developmental delay and intellectual disability [48]. The C-terminus of ESS2 (294-390 amino acids) interacts with the central domain of FRA10AC1 (96-253 amino acids) [48,49]. In the unicellular green alga *Chlamydomonas reinhardtii*, *FRA10AC1* and *ESS2* mutations affect the splicing of intraflagellar transport (*IFT*) genes [20]. IFT is an essential process in all organisms that serves to move proteins along flagella or cilia in either direction [50]. Lin et al. (2018) found splice site mutations in two *IFT* genes, *IFT81* (*fla9*) and *IFT121* (*ift121-2*), which led to flagellar assembly defects in *C. reinhardtii*. They showed that an *ESS2* (*dgr14*) deletion mutant suppressed the 3’ splice site mutation in *IFT81*, and a frameshift mutant of *FRA10* suppressed the 5’ splice site mutation in *IFT121*. Moreover, double mutants of *ESS2* and *FRA10* suppressed both splice site mutations (*IFT81* and *IFT121*) [20]. These studies indicate the important role of ESS2-mediated splicing regulation in the nervous system. Elucidating the function of ESS2 in neuronal differentiation and homeostasis remains a challenge for the future.

#### 2.5.2. ESS2 Function in T Cells

Defects of the immune system are a key feature of DiGeorge syndrome. There is also a loss of T cells with thymic defects, with 75–80% of infants with 22q11DS showing T cell depletion. Recent studies showed that LXR/RXR target genes were defective in 22q11DS patients’ T cells [51]. In T cells, ESS2 interacts with the RORγ [25], which is a master regulator for TH17 cell differentiation, expresses inflammatory cytokine IL-17, and is also involved in naive T cell maintenance [52]. The ROR-alpha, -beta, and -gamma were named based on their high sequence homology to RAR. RORs have prominent roles in regulating circadian rhythm, immune function, and neuronal development [53]. Given their central role in pathogenic TH17 cell development and function [54], and because of encouraging data from preclinical testing of ligands, the RORs have emerged as drug target candidates for the treatment of various autoimmune diseases, including MS, rheumatoid arthritis, and psoriasis [26]. We found that ESS2 associates with RORs by IP-MS and Immunoprecipitation–Western Blot experiments [25,41]. Interestingly, wild type (1–480 aa) and the C-terminal deletion mutant of ESS2 (1–400 aa) enhanced the transcriptional activity of RORγt, but the N-terminal deletion mutant of ESS2 (101–480 aa) did not [25]. Moreover, ESS2 associates with RORγt in the N-terminal region (1–200aa) [41]. These results show the ESS2-dependent transcriptional activation does not require spliceosome association. By overexpressing ESS2-Flag protein in 68-41 cells, we previously identified ESS2-interacting proteins, including chromatin remodeling factor BAZ1B (bromodomain adjacent to the zinc finger domain 1B) and serine/threonine protein kinase RSK2 (ribosomal S6 kinase 2). RSK2 is a member of the RSK family, which is phosphorylated and activated by ERK1/2 [55]. Interestingly, the pan RSK inhibitor BI-D1870 reduced IL-17A expression in primary TH17 cells [25]. BI-D1870 also showed significant inhibition in EAE model mice, in addition to suppressing TH17 cells [56]. These results indicate that RSK inhibitors may be a new target in the treatment of autoimmune diseases.

Furthermore, we generated CD4+ T cell-specific ESS2 gene knockout (Ess2^ΔCD4/ΔCD4^) mice using CD4-Cre mice and found that the number of naive T cells decreased in Ess2^ΔCD4/ΔCD4^ mice [57]. Interestingly, Ess2^ΔCD4/ΔCD4^ mice showed decreased NKT cells and increased γδT cells in the thymus and spleen. We performed RNA-seq to investigate the molecular mechanisms underlying the disruption of naive T cell maintenance with no abnormalities in cell number and found that the expression pattern of CD4+ T cells in the thymus was altered. Particularly, we found that ESS2 enhanced mRNA expression levels of Myc target genes such as *LDHA* and *Ribosomal proteins* in CD4+ T cells. The interaction between ESS2 and Myc has been reported by IP-MS [23], and we also confirmed the ESS2-Myc interaction by fluorescence immunostaining and the ESS2-dependent enhancement of Myc transcriptional activities by luciferase reporter assay. Thus, the ESS2 gene is responsible for altering the mRNA expression of an important gene cluster during T-cell differentiation. We searched for genes with altered splicing from RNA-seq data of CD4+ T cells from control and Ess2^ΔCD4/ΔCD4^ mice but did not find any genes with significant differences.

Other groups showed the binding of ESS2 to the 3’ UTR of *IFNG* and *TNF* genes in activated human primary T cells but not to the *IL2* 3’ UTR [58]. *IFNG* and *TNF* are important cytokines for T cell activation. Thus, these studies also indicate the importance of ESS2 to gene regulation in T cells.

#### 2.5.3. ESS2 Regulates the NF-κB/CHD1 Pathway in Prostate Cancer

A recent study reported that ESS2 regulated prostate cancer proliferation primarily through the NF-κB/CHD1 pathway and that ESS2 knockdown lowered histone H3K36 trimethylation levels on the target gene promoters in castration-resistant prostate cancer (CRPC) cells [59]. ESS2 tends to be highly expressed in prostate cancer patients, and expression levels of some ESS2 target genes, such as *LIF*, *WNT5A*, and *TGFB1*, were significantly correlated with ESS2 levels in prostate cancer patients. In PC3 cells, *ESS2* knockdown reduced tumor progression and mRNA expression levels of NF-κB/CHD1 target genes such as *TNF* and *IL1B*. Although the interaction between ESS2 and NF-κB/CHD1 has not been confirmed, *ESS2* knockdown reduced NF-κB/CHD1 recruitment on the *TNF* and *CCL2* promoters without reduction of NF-κB and CHD1 protein levels [59]. Moreover, ESS2 regulated *VDR* and *PPARG* expression, and their target genes were suppressed by *ESS2* knockdown. These results are the first to demonstrate the importance of ESS2 in CRPC, but there are still questions to be addressed. Firstly, it is not known which domains of ESS2 are essential for cancer proliferation. Since the C-terminus is critical for interacting with the spliceosome and the N-terminus and Es domain are associated with RORs (nuclear receptor transcription factors) [41], some ESS2 deletion mutants may answer this question. Secondly, the role of ESS2 in chromatin structure maintenance is still unclear. Since the genes targeted by ESS2 in cancer cells and T cells are different, ESS2 must regulate transcription factors in a cell-dependent manner. Interestingly, ESS2 has been shown to mediate interactions between transcription factors and lncRNAs [41]; thus, this type of regulation may be critical for ESS2 function. Considering the CRPC-specific lncRNAs that have been reported [60], we examined ESS2-dependent NF-κB/lncRNA interactions, but ESS2 did not regulate such interactions in CRPC. Moreover, ESS2 has been shown to regulate the association of NF-κB and CHD1, which is a chromatin remodeling factor that plays a pivotal role in CRPC [61]. Since ESS2 is also associated with another chromatin remodeling factor (BAZ1B [25]), ESS2-mediated regulation in cancer seems to be complex and to involve chromatin structure.

## 3. Discussion and Conclusions

In this review, we summarized the cloning of ESS2 and its functions in numerous species, from yeast to humans, as shown in Table 1. As demonstrated by the reduced viability in *ESS2*-deficient yeast [35] and the lethality at very early stages in ESS2 knockout mice [34], ESS2 plays a pivotal role in cell maintenance. However, the exact molecular mechanism of ESS2 deficiency-dependent lethality remains to be elucidated. Previous studies showed that ESS2 interacted with numerous factors, such as transcription factors, splicing factors, and chromatin remodeling factors. Furthermore, ESS2 target genes differ among cell types, indicating that the ESS2-dependent gene expression regulation may be cell type-selective. Such cell type selective function of ESS2 may be due to the different expression of ESS2 interacting factors.

Several studies showed that the C-terminal region of ESS2 is critical for interacting with spliceosome complexes [22,41]. This region also showed a high IDR score, indicating the absence of a defined 3D structure despite being an essential region for cellular processes, ranging from transcriptional control and cell signaling to subcellular organization [62]. Future experiments are needed to prove that the C-terminus of ESS2 is an IDR, in accordance with the Minimum Information About Disorder Experiments (MIADE) [63].

Recent work has introduced the concept of “nondomain biopolymers” to describe proteins that lack classical folded domains yet orchestrate vital functions [64]. IDRs in splicing-associated factors—including ESS2 and Fam32a [18]—exemplify how these flexible segments enable the multivalent, transient interactions essential for the dynamic assembly and rearrangement of spliceosomal components. It will be important to clarify how such “nondomain biopolymers” regulate molecular condensates and other macromolecular complexes, such as spliceosomes, possibly by modulating these multivalent interactions. Understanding these mechanisms could not only unveil fundamental aspects of splicing biology but also provide insight into novel modalities that expand the functional capabilities of these complexes.

Regarding the ESS2-dependent regulation of transcription factors, it is also possible that ESS2 mediates splicing control, but the molecular mechanism has not yet been elucidated. The N-terminal region of ESS2 requires RORγt interaction and transcriptional activation, while ESS2 associates spliceosome in the C-terminal region [25,41]. Thus, the ESS2-dependent regulation of transcription and splicing may be different mechanisms. Since ESS2 regulates histone modifications involved in transcription elongation rather than in initiation [59], analyzing the ESS2 behavior in dynamic states during transcription and splicing will be necessary for future studies. There are also still remaining questions regarding the molecular mechanism of ESS2 in the splicing process. For example, the selectivity of ESS2 target mRNAs and the role of ESS2 in the C and C* complex are still unknown. Recently, the interaction between U1 snRNP and ESS2, which is not included in the C complex, has also been reported [16,41]. Because U1 snRNP suppresses premature 3′-end cleavage and polyadenylation for inhibiting aberrant transcription termination [65,66], this result suggests that ESS2 may also regulate mRNA metabolism during transcription.

ESS2 interacts with several transcription factors, especially with nuclear receptors (NRs), such as RORs, NR4A, and TR2. NRs are transcriptional factors directly bound with lipophilic hormones that regulate mRNA expression [27]. In general, NRs share a common structural organization that defines this gene superfamily, which consists of an N-terminal domain containing an activation function 1 (AF1) domain, a DNA binding domain (DBD) consisting of C4 type zinc finger domain, a hinge region, and a ligand binding domain (LBD), which associates transcriptional coregulators through its activation function 2 (AF2) domain. Interestingly, RORγ/γt associates with ESS2 in the hinge region [25]. Since the transcriptional coregulators bound to the hinge region are rare, ESS2-dependent transcriptional regulation may be different from other coregulators. Because ESS2 overexpression enhances the transcriptional activities of RORγ/γt [25] and Myc [57], the function of ESS2 in transcription must differ from its function in splicing. The different functions of the ESS2 protein in transcription and splicing remain unclear, but post-translational modification of ESS2 may be the key to distinguishing the use of transcription factors from splicing. Although ESS2 has been shown to have modification potential, such as in phosphorylation, its function has not been reported. In particular, the C-terminal region of ESS2 contains several conserved MAP kinase phosphorylation sites (Ser/Thr-Phe) despite the low amino acid conservation. Thus, future studies will be needed.

In summary, ESS2 plays an important role in cell differentiation/proliferation through the regulation of transcription and splicing, but its molecular mechanism is still unclear. Moreover, ESS2 may have a potential for a biomarker and therapeutic target, particularly in cancer, autoimmune disease, and neurodevelopmental disorders. Further studies will provide new findings and lead to the establishment of new therapies for cancer and neural and immune diseases.

## Figures and Tables

**Table 1:** ESS2 functions, interacting proteins, and regulations.

Species	Functions	Interacting Proteins, Regulatory Factors	Ref.
Yeast	ESS2 overexpression; Cell elongation phenotype	Interact with ISH1, PRMT5	[35]
	ESS2 deficient; reduce viability		
Green alga	IFT mutation splicing regulation	Regulate splicing with FRA10AC1	[20]
Arabidopsis	Mutnants (Q80* and W365*) defect Pre-mRNA splicing		[21]
	Salt sress-dependent mRNA splicing	Regulate SWI3 mRNA expression	[22]
*C. elegans*	Neuron development		[19]
Drosophila	Fat cells regulation		[43]
Mouse	CD4+ naive T cell maintenance, TH17 differentiation	Regulate mRNA expression and transcriptional activities of RORg/gt and Myc	[25,57]
	Neuron	Regulate mRNA expression	[46]
Human	Prostate cancer proliferation	Regulate such as NF-kB/CHD1-dependent mRNA expression	[59]
	Neuron	Interact with FRA10AC1, mRNA and splicing regulation	[48,49]
	Dopamin neuron differentiation from iPS	PERK protein expression	[45]

## References

[1] Gong W., Emanuel B.S., Collins J., Kim D.H., Wang Z., Chen F., Zhang G., Roe B., Budarf M.L. (1996). A transcription map of the DiGeorge and velo-cardio-facial syndrome minimal critical region on 22q11. Hum. Mol. Genet..

[2] Lindsay E.A., Rizzu P., Antonacci R., Jurecic V., Delmas-Mata J., Lee C.C., Kim U.J., Scambler P.J., Baldini A. (1996). A transcription map in the CATCH22 critical region: Identification, mapping, and ordering of four novel transcripts expressed in heart. Genomics.

[3] Scambler P.J. (2000). The 22q11 deletion syndromes. Hum. Mol. Genet..

[4] Robin N.H., Shprintzen R.J. (2005). Defining the clinical spectrum of deletion 22q11.2. J. Pediatr..

[5] McDonald-McGinn D.M., Sullivan K.E. (2011). Chromosome 22q11.2 deletion syndrome (DiGeorge syndrome/velocardiofacial syndrome). Medicine.

[6] Sullivan K.E. (2019). Chromosome 22q11.2 deletion syndrome and DiGeorge syndrome. Immunol. Rev..

[7] Lindsay E.A., Halford S., Wadey R., Scambler P.J., Baldini A. (1993). Molecular cytogenetic characterization of the DiGeorge syndrome region using fluorescence in situ hybridization. Genomics.

[8] Rizzu P., Lindsay E.A., Taylor C., O’Donnell H., Levy A., Scambler P., Baldini A. (1996). Cloning and comparative mapping of a gene from the commonly deleted region of DiGeorge and Velocardiofacial syndromes conserved in *C. elegans*. Mamm. Genome.

[9] Gong W., Emanuel B.S., Galili N., Kim D.H., Roe B., Driscoll D.A., Budarf M.L. (1997). Structural and mutational analysis of a conserved gene (DGSI) from the minimal DiGeorge syndrome critical region. Hum. Mol. Genet..

[10] Lindsay E.A., Harvey E.L., Scambler P.J., Baldini A. (1998). ES2, a gene deleted in DiGeorge syndrome, encodes a nuclear protein and is expressed during early mouse development, where it shares an expression domain with a Goosecoid-like gene. Hum. Mol. Genet..

[11] Guna A., Butcher N.J., Bassett A.S. (2015). Comparative mapping of the 22q11.2 deletion region and the potential of simple model organisms. J. Neurodev. Disord..

[12] Herold N., Will C.L., Wolf E., Kastner B., Urlaub H., Luhrmann R. (2009). Conservation of the protein composition and electron microscopy structure of Drosophila melanogaster and human spliceosomal complexes. Mol. Cell Biol..

[13] Bessonov S., Anokhina M., Will C.L., Urlaub H., Luhrmann R. (2008). Isolation of an active step I spliceosome and composition of its RNP core. Nature.

[14] Hegele A., Kamburov A., Grossmann A., Sourlis C., Wowro S., Weimann M., Will C.L., Pena V., Luhrmann R., Stelzl U. (2012). Dynamic protein-protein interaction wiring of the human spliceosome. Mol. Cell.

[15] Kastner B., Will C.L., Stark H., Luhrmann R. (2019). Structural Insights into Nuclear pre-mRNA Splicing in Higher Eukaryotes. Cold Spring Harb. Perspect. Biol..

[16] Dybkov O., Preussner M., El Ayoubi L., Feng V.Y., Harnisch C., Merz K., Leupold P., Yudichev P., Agafonov D.E., Will C.L. (2023). Regulation of 3′ splice site selection after step 1 of splicing by spliceosomal C* proteins. Sci. Adv..

[17] Zhan X., Lu Y., Zhang X., Yan C., Shi Y. (2022). Mechanism of exon ligation by human spliceosome. Mol. Cell.

[18] Fica S.M., Oubridge C., Wilkinson M.E., Newman A.J., Nagai K. (2019). A human postcatalytic spliceosome structure reveals essential roles of metazoan factors for exon ligation. Science.

[19] Noma K., Goncharov A., Jin Y. (2014). Systematic analyses of rpm-1 suppressors reveal roles for ESS-2 in mRNA splicing in Caenorhabditis elegans. Genetics.

[20] Lin H., Zhang Z., Iomini C., Dutcher S.K. (2018). Identifying RNA splicing factors using IFT genes in Chlamydomonas reinhardtii. Open Biol..

[21] Kanno T., Venhuizen P., Wu M.T., Chiou P., Chang C.L., Kalyna M., Matzke A.J.M., Matzke M. (2020). A Collection of Pre-mRNA Splicing Mutants in Arabidopsis thaliana. G3 Genes Genomes Genet..

[22] Xie M., Tadesse D., Zhang J., Yao T., Zhang L., Jawdy S.S., Devireddy A., Zheng K., Smith E.B., Morrell-Falvey J. (2024). AtDGCR14L contributes to salt-stress tolerance via regulating pre-mRNA splicing in Arabidopsis. Plant J..

[23] Kalkat M., Resetca D., Lourenco C., Chan P.K., Wei Y., Shiah Y.J., Vitkin N., Tong Y., Sunnerhagen M., Done S.J. (2018). MYC Protein Interactome Profiling Reveals Functionally Distinct Regions that Cooperate to Drive Tumorigenesis. Mol. Cell.

[24] Goos H., Kinnunen M., Salokas K., Tan Z., Liu X., Yadav L., Zhang Q., Wei G.H., Varjosalo M. (2022). Human transcription factor protein interaction networks. Nat. Commun..

[25] Takada I. (2015). DGCR14 induces Il17a gene expression through the RORgamma/BAZ1B/RSKS2 complex. Mol. Cell Biol..

[26] Yang P.B., Hou P.P., Liu F.Y., Hong W.B., Chen H.Z., Sun X.Y., Li P., Zhang Y., Ju C.Y., Luo L.J. (2020). Blocking PPARgamma interaction facilitates Nur77 interdiction of fatty acid uptake and suppresses breast cancer progression. Proc. Natl. Acad. Sci. USA.

[27] Evans R.M., Mangelsdorf D.J. (2014). Nuclear Receptors, RXR, and the Big Bang. Cell.

[28] Burris T.P., de Vera I.M.S., Cote I., Flaveny C.A., Wanninayake U.S., Chatterjee A., Walker J.K., Steinauer N., Zhang J., Coons L.A. (2023). International Union of Basic and Clinical Pharmacology CXIII: Nuclear Receptor Superfamily-Update 2023. Pharmacol. Rev..

[29] Yu L., Jearawiriyapaisarn N., Lee M.P., Hosoya T., Wu Q., Myers G., Lim K.C., Kurita R., Nakamura Y., Vojtek A.B. (2018). BAP1 regulation of the key adaptor protein NCoR1 is critical for gamma-globin gene repression. Genes. Dev..

[30] DiBlasi E., Shabalin A.A., Monson E.T., Keeshin B.R., Bakian A.V., Kirby A.V., Ferris E., Chen D., William N., Gaj E. (2021). Rare protein-coding variants implicate genes involved in risk of suicide death. Am. J. Med. Genet. B Neuropsychiatr. Genet..

[31] Hoogendoorn B., Coleman S.L., Guy C.A., Smith S.K., O’Donovan M.C., Buckland P.R. (2004). Functional analysis of polymorphisms in the promoter regions of genes on 22q11. Hum. Mutat..

[32] Wang H., Duan S., Du J., Li X., Xu Y., Zhang Z., Wang Y., Huang G., Feng G., He L. (2006). Transmission disequilibrium test provides evidence of association between promoter polymorphisms in 22q11 gene DGCR14 and schizophrenia. J. Neural Transm..

[33] Suh J.H., Yoon J.S., Kwon J.B., Kim H.W., Wang Y.P. (2011). Identification of genomic aberrations by array comparative genomic hybridization in patients with aortic dissections. Korean J. Thorac. Cardiovasc. Surg..

[34] Lindsay E.A., Botta A., Jurecic V., Carattini-Rivera S., Cheah Y.C., Rosenblatt H.M., Bradley A., Baldini A. (1999). Congenital heart disease in mice deficient for the DiGeorge syndrome region. Nature.

[35] Taricani L., Tejada M.L., Young P.G. (2002). The fission yeast ES2 homologue, Bis1, interacts with the Ish1 stress-responsive nuclear envelope protein. J. Biol. Chem..

[36] Sengupta S., Kennemer A., Patrick K., Tsichlis P., Guerau-de-Arellano M. (2020). Protein Arginine Methyltransferase 5 in T Lymphocyte Biology. Trends Immunol..

[37] Zheng J., Li B., Wu Y., Wu X., Wang Y. (2023). Targeting Arginine Methyltransferase PRMT5 for Cancer Therapy: Updated Progress and Novel Strategies. J. Med. Chem..

[38] Genau A.C., Li Z., Renzaglia K.S., Fernandez Pozo N., Nogue F., Haas F.B., Wilhelmsson P.K.I., Ullrich K.K., Schreiber M., Meyberg R. (2021). HAG1 and SWI3A/B control of male germ line development in P. patens suggests conservation of epigenetic reproductive control across land plants. Plant Reprod..

[39] Sheta M., Yoshida K., Kanemoto H., Calderwood S.K., Eguchi T. (2023). Stress-Inducible SCAND Factors Suppress the Stress Response and Are Biomarkers for Enhanced Prognosis in Cancers. Int. J. Mol. Sci..

[40] Huang W.P., Ellis B.C.S., Hodgson R.E., Sanchez Avila A., Kumar V., Rayment J., Moll T., Shelkovnikova T.A. (2024). Stress-induced TDP-43 nuclear condensation causes splicing loss of function and STMN2 depletion. Cell Rep..

[41] Takada I., Tsuchiya M., Yanaka K., Hidano S., Takahashi S., Kobayashi T., Ogawa H., Nakagawa S., Makishima M. (2018). Ess2 bridges transcriptional regulators and spliceosomal complexes via distinct interacting domains. Biochem. Biophys. Res. Commun..

[42] Nakata K., Abrams B., Grill B., Goncharov A., Huang X., Chisholm A.D., Jin Y. (2005). Regulation of a DLK-1 and p38 MAP kinase pathway by the ubiquitin ligase RPM-1 is required for presynaptic development. Cell.

[43] Baumbach J., Hummel P., Bickmeyer I., Kowalczyk K.M., Frank M., Knorr K., Hildebrandt A., Riedel D., Jackle H., Kuhnlein R.P. (2014). A Drosophila in vivo screen identifies store-operated calcium entry as a key regulator of adiposity. Cell Metab..

[44] Toprak U., Hegedus D., Dogan C., Guney G. (2020). A journey into the world of insect lipid metabolism. Arch. Insect Biochem. Physiol..

[45] Arioka Y., Shishido E., Kushima I., Suzuki T., Saito R., Aiba A., Mori D., Ozaki N. (2021). Chromosome 22q11.2 deletion causes PERK-dependent vulnerability in dopaminergic neurons. EBioMedicine.

[46] Santinha A.J., Klingler E., Kuhn M., Farouni R., Lagler S., Kalamakis G., Lischetti U., Jabaudon D., Platt R.J. (2023). Transcriptional linkage analysis with in vivo AAV-Perturb-seq. Nature.

[47] Bessonov S., Anokhina M., Krasauskas A., Golas M.M., Sander B., Will C.L., Urlaub H., Stark H., Luhrmann R. (2010). Characterization of purified human Bact spliceosomal complexes reveals compositional and morphological changes during spliceosome activation and first step catalysis. RNA.

[48] von Elsner L., Chai G., Schneeberger P.E., Harms F.L., Casar C., Qi M., Alawi M., Abdel-Salam G.M.H., Zaki M.S., Arndt F. (2022). Biallelic FRA10AC1 variants cause a neurodevelopmental disorder with growth retardation. Brain.

[49] Sarafidou T., Galliopoulou E., Apostolopoulou D., Fragkiadakis G.A., Moschonas N.K. (2023). Reconstruction of a Comprehensive Interactome and Experimental Data Analysis of FRA10AC1 May Provide Insights into Its Biological Role in Health and Disease. Genes.

[50] Gupta M., Pazour G.J. (2024). Intraflagellar transport: A critical player in photoreceptor development and the pathogenesis of retinal degenerative diseases. Cytoskeleton.

[51] Raje N.R., Noel-MacDonnell J.R., Shortt K.A., Gigliotti N.M., Chan M.A., Heruth D.P. (2022). T Cell Transcriptome in Chromosome 22q11.2 Deletion Syndrome. J. Immunol..

[52] Capone A., Volpe E. (2020). Transcriptional Regulators of T Helper 17 Cell Differentiation in Health and Autoimmune Diseases. Front. Immunol..

[53] Jetten A.M. (2009). Retinoid-related orphan receptors (RORs): Critical roles in development, immunity, circadian rhythm, and cellular metabolism. Nucl. Recept. Signal.

[54] Huang W., Littman D.R. (2015). Regulation of RORgammat in Inflammatory Lymphoid Cell Differentiation. Cold Spring Harb. Symp. Quant. Biol..

[55] Spirrison A.N., Lannigan D.A. (2024). RSK1 and RSK2 as therapeutic targets: An up-to-date snapshot of emerging data. Expert. Opin. Ther. Targets.

[56] Takada I., Yogiashi Y., Makishima M. (2016). The ribosomal S6 kinase inhibitor BI-D1870 ameliorated experimental autoimmune encephalomyelitis in mice. Immunobiology.

[57] Takada I., Hidano S., Takahashi S., Yanaka K., Ogawa H., Tsuchiya M., Yokoyama A., Sato S., Ochi H., Nakagawa T. (2022). Transcriptional coregulator Ess2 controls survival of post-thymic CD4(+) T cells through the Myc and IL-7 signaling pathways. J. Biol. Chem..

[58] Popovic B., Nicolet B.P., Guislain A., Engels S., Jurgens A.P., Paravinja N., Freen-van Heeren J.J., van Alphen F.P.J., van den Biggelaar M., Salerno F. (2023). Time-dependent regulation of cytokine production by RNA binding proteins defines T cell effector function. Cell Rep..

[59] Takahashi S., Takada I., Hashimoto K., Yokoyama A., Nakagawa T., Makishima M., Kume H. (2023). ESS2 controls prostate cancer progression through recruitment of chromodomain helicase DNA binding protein 1. Sci. Rep..

[60] Takayama K.I., Fujimura T., Suzuki Y., Inoue S. (2020). Identification of long non-coding RNAs in advanced prostate cancer associated with androgen receptor splicing factors. Commun. Biol..

[61] Zhao D., Lu X., Wang G., Lan Z., Liao W., Li J., Liang X., Chen J.R., Shah S., Shang X. (2017). Synthetic essentiality of chromatin remodelling factor CHD1 in PTEN-deficient cancer. Nature.

[62] Holehouse A.S., Kragelund B.B. (2024). The molecular basis for cellular function of intrinsically disordered protein regions. Nat. Rev. Mol. Cell Biol..

[63] Meszaros B., Hatos A., Palopoli N., Quaglia F., Salladini E., Van Roey K., Arthanari H., Dosztanyi Z., Felli I.C., Fischer P.D. (2023). Minimum information guidelines for experiments structurally characterizing intrinsically disordered protein regions. Nat. Methods.

[64] Arakawa K., Hirose T., Inada T., Ito T., Kai T., Oyama M., Tomari Y., Yoda T., Nakagawa S. (2023). Nondomain biopolymers: Flexible molecular strategies to acquire biological functions. Genes. Cells.

[65] So B.R., Di C., Cai Z., Venters C.C., Guo J., Oh J.M., Arai C., Dreyfuss G. (2019). A Complex of U1 snRNP with Cleavage and Polyadenylation Factors Controls Telescripting, Regulating mRNA Transcription in Human Cells. Mol. Cell.

[66] Venters C.C., Oh J.M., Di C., So B.R., Dreyfuss G. (2019). U1 snRNP Telescripting: Suppression of Premature Transcription Termination in Introns as a New Layer of Gene Regulation. Cold Spring Harb. Perspect. Biol..

